# The role of digital clinical decision support tool in improving quality of intrapartum and postpartum care: experiences from two states of India

**DOI:** 10.1186/s12884-021-03710-y

**Published:** 2021-04-07

**Authors:** Gulnoza Usmanova, Kamlesh Lalchandani, Ashish Srivastava, Chandra Shekhar Joshi, Deepak Chandra Bhatt, Anand Kumar Bairagi, Yashpal Jain, Mohammed Afzal, Rashmi Dhoundiyal, Jyoti Benawri, Tarun Chaudhary, Archana Mishra, Rajni Wadhwa, Pompy Sridhar, Nupur Bahl, Pratibha Gaikwad, Bulbul Sood

**Affiliations:** 1 Jhpiego-An Affiliate of Johns Hopkins University, New Delhi, 110020 India; 2 Department of Health and Family Welfare, NHM, Jaipur, Rajasthan 302001 India; 3 Maternal Health, NHM, Bhopal, Madhya Pradesh 462011 India; 4 Project Management Unit, ASMAN: Alliance for Saving Mothers and Newborns, Mumbai, 400021 India; 5 MSD for Mothers, Mumbai, 4000098 India; 6 Reliance Foundation, Mumbai, 400021 India; 7 Tata Trusts, Mumbai, 400005 India

**Keywords:** Intrapartum care, Postpartum care, Maternal health, New-born health, CDSS, mHealth, Health information technology, Quality improvement

## Abstract

**Background:**

Computerized clinical decision support (CDSS) –digital information systems designed to improve clinical decision making by providers – is a promising tool for improving quality of care. This study aims to understand the uptake of ASMAN application (defined as completeness of electronic case sheets), the role of CDSS in improving adherence to key clinical practices and delivery outcomes.

**Methods:**

We have conducted secondary analysis of program data (government data) collected from 81 public facilities across four districts each in two sates of Madhya Pradesh and Rajasthan. The data collected between August –October 2017 (baseline) and the data collected between December 2019 – March 2020 (latest) was analysed. The data sources included: digitized labour room registers, case sheets, referral and discharge summary forms, observation checklist and complication format. Descriptive, univariate and multivariate and interrupted time series regression analyses were conducted.

**Results:**

The completeness of electronic case sheets was low at postpartum period (40.5%), and in facilities with more than 300 deliveries a month (20.9%). In multivariate logistic regression analysis, the introduction of technology yielded significant improvement in adherence to key clinical practices. We have observed reduction in fresh still births rates and asphyxia, but these results were not statistically significant in interrupted time series analysis. However, our analysis showed that identification of maternal complications has increased over the period of program implementation and at the same time referral outs decreased.

**Conclusions:**

Our study indicates CDSS has a potential to improve quality of intrapartum care and delivery outcome. Future studies with rigorous study design is required to understand the impact of technology in improving quality of maternity care.

**Supplementary Information:**

The online version contains supplementary material available at 10.1186/s12884-021-03710-y.

## Introduction

Globally, it has been proven that effective and quality of care at the facility level, particularly around childbirth and immediately after the birth, can significantly contribute to the reduction of maternal deaths, stillbirths and neonatal deaths [1, 2]. Over the last decades, efforts in ensuring skilled birth attendance resulted in increased institutional deliveries, this in turn moved higher proportion of avoidable maternal and neonatal mortality to health facilities [2]. Therefore, improving quality of care around childbirth and immediately after birth is imperative to prevent adverse outcomes for pregnant women and new-borns [2].

In India, despite a tremendous increase in rate of institutional deliveries [3], maternal and neonatal deaths did not show equivalent reduction [4] in the last decade. It was found by few studies that the increase in the rate of institutional deliveries was not matched with overall improvement in quality of maternity care [4–6]. Quality of care is a multi-dimensional concept that incudes availability of evidence based guidelines, strengthened infrastructure, resources, enabling environment, attitude of health providers; all these in turn result in patients’ and providers’ satisfaction and improved health outcomes [2]. To address these issues the, Government of India (GoI), launched the quality improvement initiative under the name ‘Dakshata’ (means adroitness) [7], to build provider adherence to key evidence based clinical practices during intrapartum and immediate postpartum periods. Dakshata uses the World Health Organization’s Safe Childbirth Checklist (SCC) as a framework for improving providers’ competency. Although, Dakshata intervention has not been evaluated, other quality of intrapartum care improvement initiatives using SCC in Rajasthan [8], Uttar Pradesh [9] and Karnataka [10] demonstrated significant improvement in adherence to evidence based clinical practices. Moreover, a study from Rajasthan showed reduction in perinatal mortality, which fell by 11% [11]. However, these studies detected certain clinical practices that either did not improve significantly (initiation of partograph, counselling on danger signs), or declined over 12 months’ period (use of SCC, administration of oxytocin soon after delivery). Thus, it is imperative to identify and test innovative solutions for improving quality of care in order to accelerate reduction in maternal and neonatal death in India. One such solution is computerized clinical decision support (CDSS) –digital information systems designed to improve clinical decision making by providers – a promising tool for improving quality of care by improving adherence to clinical guidelines [12, 13], practitioner’s performance [12, 14], patient outcomes [15–17], and quality of clinical documentation [18], thus contributing to quality improvement and overall efficiency of health care delivery. While there is significant research on CDSSs generally, but evidence of use of CDSS for maternity care is limited. In rural health facilities in Burkina Faso, Ghana and Tanzania, CDSS was not found to significantly improve quality of antenatal and delivery care [19]. A study conducted in South Africa showed that CDSS lead to overall improvement in adherence to clinical guidelines, but it was not statistically significant [20]. In Nigeria, McNabb et al., reported that decision support via mobile phones led to significant improvement in health counselling, technical services, quality of health education and patient satisfaction [21].

### ASMAN intervention

In this paper we describe the findings from implementation of “ASMAN” (The Alliance for Saving Mothers and Newborns) a provider-focused package of interventions that leverages technology to reduce maternal and early neonatal mortality through the adoption of key technologies that improve capacity-building and service delivery efforts focused on the provision of quality care during childbirth and the first 48–60 h after delivery.

The ASMAN project takes an integrated quality improvement approach:

1. Competency building – through provision of training for Medical Officers (MOs), Staff Nurses, Auxiliary Nurse Midwife (ANM) on clinical skills and quality of care as per the established labour room protocols and standards. For this purpose, the project adopted GoI approved Dakshata training package for building the capacity of service providers. Post training follow up and on-site mentoring were conducted by the program team to ensure translation of skills into practice.
2. Introduction of technology intervention to facilitate timely and correct clinical decision-making by providers. For this purpose, ASMAN, an android based application for electronic recording of intrapartum and immediate postpartum care, integrated with Clinical Decision Support, e-partograph, knowledge enhancement and other features was introduced at project sites. The ASMAN application runs on a tablet stationed in several important areas of the health facilities, including registration, triage, the labor room, postpartum areas, operation theatre and post-operative wards.

The ASMAN application had the following components:

- Case management: Digitized case sheet from admission until discharge with integrated clinical rules (admission notes, e-partogram, Safe Childbirth Checklist, delivery notes, post-delivery monitoring, post-natal care, discharge slip, referral slip, events section, alerts & notifications).
- Dashboards and reports: System generated dashboards and reports for respective health facility, district and state level managers.
- E-learning content: All GoI training modules, guidelines and tutorials. Available in audio, video, or readable format in English or in Hindi
- ASMAN Complication Management Game: a case-based game designed to improve management of intrapartum and immediate postpartum/ postnatal complications for developing critical thinking skills of health workers around safe child delivery
- Safe Delivery App: which provides evidence-based clinical guidelines on Basic Emergency Obstetric and Neonatal Care
- Remote support center: staffed 24/7 by senior residents at the medical college for provision of support in cases of unclear management. Staff at the remote support center have access to all cases

ASMAN program implementation took place across 81 public facilities in four districts each in Rajasthan and Madhya Pradesh states of India. The key stakeholders of ASMAN were Governments of Madhya Pradesh and Rajasthan. This project was piloted between June 2017 to May 2020, with support from Reliance Foundation, Tata Trusts, MSD for Mothers, Bill and Melinda Gates Foundation and United States Agency for International Development (USAID). Jhpiego was the lead implementation agency. The respective state governments were consulted to select specific intervention districts that had higher neonatal and maternal mortality rates as compared to the state average, districts without ongoing intervention for improving quality of intrapartum and postpartum care. Within those districts, government health facilities that had a relatively high case load of 50 or more deliveries per month and therefore, greater need for intervention, were selected.

This program implementation offered an opportunity to evaluate the role of a CDSS in improving quality of care during the intrapartum and immediate postpartum periods, assess its impact on delivery outcomes, identification and referral of key maternal and neonatal complications in selected public facilities.

## Methodology

### Research aims

To our knowledge, no studies from India have described the impact of CDSS on clinical outcomes during the intrapartum and immediate postpartum periods. Along with the lack of evidence on effect of CDSS in improving quality of maternity care, this gap represents a crucial research priority for India with global relevance. Through this analysis, we attempt to answer the following questions:

1. What was the uptake of ASMAN application among health care providers?
2. How did the ASMAN application affect adherence to key clinical practices?
3. What was the trend of maternal (pre-eclampsia, eclampsia, postpartum haemorrhage) and neonatal (fresh still births, birth asphyxia) complications during the project implementation?
4. What was the trend of identification and referral of maternal complications (pre-eclampsia, eclampsia, postpartum haemorrhage) during the project implementation?

### Study design, and study setting

We have conducted secondary analysis of program data (government data) collected from 81 public facilities across four districts each in two states of Madhya Pradesh (Jabalpur, Khargone, Ratlam and Vidisha) and Rajasthan (Ajmer, Bhilwara, Kota and Jhalawar). This de-identified dataset was provided by the government of both states. The data collected between August –October 2017 was considered as a baseline and the data collected between December 2019 – March 2020 was considered as a latest assessment. The study was performed in accordance with ethical principles outlined in the World Medical Association (WMA) Declaration of Helsinki [22] where privacy and confidentiality of personal information was assured by getting access to de-identified data.

### Study tools and data collection

The following tools were used to collect data:

- ASMAN application data- has digitized all labour room registers, case sheets, referral and discharge summary forms of Government of India. Every woman who comes to any ASMAN facility for delivery is registered in the application. All forms are filled by the health providers at the 81 project facilities in real time. Details from patient history, labour room case sheets, partograph, until discharge is collected in the ASMAN application. We have used data from ASMAN application to understand filling ratio at admission, delivery, post-delivery, postnatal care (PNC) and discharge. Data of all those women who had delivered at ASMAN facilities and their newborns between November 2017 and March 2020 in Rajasthan and Madhya Pradesh states are used for this analysis. Till March 2020, data of 266,992 women who delivered and 228,807 newborns were entered in ASMAN application.

- The observation checklist was developed based on WHO SCC [23] and was approved by GoI as part of monitoring activities under Dakshata program [24]. This checklist was designed to measure adherence to essential practices around childbirth. These practices were divided by pause points: practices that are necessary to perform on admission, just before pushing (or before caesarean), soon after birth (1 h) and before discharge. This tool was used to explore adherence to practices at the baseline and latest assessment periods. The program officers, with medical background, collected this information; the observation assessment on average took 2–4 h.
- Complication format: Maternal and New Born Complications Identification and Referral Monitoring System. Since, there is no formal mechanism in the government systems to monitor childbirth and new born related complication and management, complication format was introduced by ASMAN program team with the support from the Governments of Madhya Pradesh and Rajasthan. The complication reporting format captured data on key maternal and newborn health indicators from the labour room, postpartum ward, Sick Newborn Care Unit (SNCU), admissions and discharge department of the facility. This format captured data on total deliveries, type of deliveries, fresh stillbirths, preterm births, maternal and neonatal death, maternal complications (pre-eclampsia, eclampsia, sepsis, postpartum haemorrhage, sepsis), neonatal complications (neonatal asphyxia and sepsis), refer in and refer out data for maternal and neonatal complications. One nodal person, preferably a labour room staff, was selected from each intervention facility to act as the key contact person for this initiative and was validated regularly by ASMAN program team. This tool was used to explore effect of ASMAN application on delivery outcomes.

### Data analysis

To describe uptake of ASMAN application, we computed the filling rates of 75 key data fields of the application and analysed the differences by pause points, level of health facility and average annual delivery load at the health facilities.

To analyze the change in adherence to evidence based practices after roll out of the intervention, we combined the baseline assessment and last periodic assessment data sets. For overall univariate analysis of adherence to key clinical practices before and after comparison, we computed means for before and latest intervention periods and compared them using chi square test. For adherence to evidence based practices, we also performed multivariable logistic regression analysis for each practice while adjusting for level of facility, average delivery load at the facility and availability of resources required for each key practice.

To analyze trends in maternal and neonatal complications, and referrals we utilized the monthly complication format data which was shared by all ASMAN intervention facilities. As the intervention was rolled out in a staggered manner at different time points across project health facilities, we categorized the complication data of each facility into two time periods corresponding to before and after intervention roll out.

Within each time period, we further grouped monthly data into quarterly data (3 months’ data), such that each quarter represented a time interval relative to the intervention roll out. For example, Q-1 and Q + 1 denoted the periods corresponding to 3 months before and 3 months after intervention rollout respectively. The month of intervention roll out together with subsequent 2 months were denoted as Q0 or quarter 0 or the time interval corresponding to intervention roll out. Data of corresponding quarters was combined for all facilities and this dataset was used for generating simple time series plots (line/bar) for analysing the trends before and after intervention roll out.

Single group interrupted time series regression analysis was performed on two dependent variables 1) fresh still birth rate and 2) incidence of neonatal asphyxia. We have conducted interrupted time series analysis for these two outcomes as these were primary outcomes of the intervention. Monthly data was utilized for carrying out this analysis, yielding data for 12 time points before and after intervention roll out respectively (M − 12 to M-1 before roll out and M + 1 to M + 12 after roll out). Coefficients or parameters which represented a shift in the level (intercept) of the dependent variable and a shift in the rate of change (slope) of dependent variable, after intervention, were computed along with 95% confidence intervals. Newey west standard errors were utilized to account for autocorrelation.

For all statistical tests of significance, *p* value < 0.05 was considered significant. We used Statistics package for the Social Sciences (SPSS), version 24, Stata version 14 and Microsoft Excel, for data analysis.

## Results

### Characteristics of facilities

Sixty-three percent of 81 intervention facilities were community health centres. More than half of intervention facilities had average monthly delivery load less than 100 (Table 1).

**Table 1:** Characteristics of ASMAN facilities

Characteristics	Total number	Percent
**Facility type**		
District Hospital	**6**	7.4
Sub-divisional (district) Hospitals	**11**	13.6
Satellite hospital	**1**	1.2
Community Health Centre	**51**	63.0
Primary Health Centre	**12**	14.8
**Monthly delivery load**		
Less than 100	**41**	50.6
100–200	**27**	33.3
201–300	**5**	6.2
Above 300	**8**	9.9
**Total**	**81**	**100**

### Uptake of ASMAN application

The analysis of filling ratio of ASMAN application revealed that filling ratio was lowest at the PNC period (40.5%), followed by admission (80.2%), discharge (82.9%), post-delivery (93.1%) and delivery (93.7%) (Table 3). Further, it was also found that filling ratio was low in high delivery load facilities compare to low and medium delivery load facilities (Table 2). However, filling ratio was not statistically significant by facility type and by delivery load.

**Table 2:** Filling Ratio of key indicators at different pause points by facility type and delivery load

**By facility type**						
**Facility Type**	**Admission**	**Delivery**	**Post delivery**	**PNC**	**Discharge**	***P*** **value**
District Hospital (*n* = 6)	69.8%	91.1%	88.3%	23.2%	82.4%	0.08
Sub-divisional (district) Hospitals (*n* = 11)	84.6%	95.1%	93.0%	42.4%	83.3%	
Satellite Hospital (*n* = 1)	92.0%	97.5%	96.5%	74.7%	89.1%	
Community Health Centre (*n* = 51)	87.3%	95.3%	97.2%	50.7%	82.2%	
Primary Health Centre (*N* = 12)	90.7%	96.1%	98.6%	73.3%	86.5%	
**Grand Total (*N*** **= 81)**	**80.2%**	**93.7%**	**93.1%**	**40.5%**	**82.9%**	
**By delivery load**						
**Delivery load**	**Admission**	**Delivery**	**Post delivery**	**PNC**	**Discharge**	***P*** **value**
Less than 100 (*n* = 41)	91.0%	96.0%	97.3%	68.5%	84.9%	0.05
100–200 (*n* = 27)	87.8%	95.1%	97.7%	51.4%	82.6%	
201–300 (*n* = 5)	85.4%	96.5%	97.9%	47.4%	87.2%	
above 300 (*n* = 8)	70.3%	91.3%	87.7%	20.9%	81.3%	
**Grand Total (*n*** **= 81)**	**80.2%**	**93.7%**	**93.1%**	**40.5%**	**82.9%**	

### Adherence to key clinical practices

Table 3 illustrates providers’ adherence to key clinical practices across all pause points at the baseline and latest assessment. In univariate analysis, statistically significant improvement in adherence to key clinical practices was observed in 18 of 20 key clinical practices. Measuring birth weight and breastfeeding initiation were the only practices that did not show statistically significant change in univariate analysis; both of these practices were above 70% at the time of baseline assessment. However, after adjusting for level of facility, average annual delivery load and resource availability all clinical practices showed significant improvement (Table 3).

**Table 3:** Univariate and multivariate analysis of providers’ adherence to key clinical practices (%)

Practices	Baseline (August –October 2017)	Latest (December 2019 – March 2020)	***p***-value (univariate analysis)	***p*** value (multivariate^**a**^ analysis)
**On admission**				
Records fetal heart rate at admission	46	96	0.004	< 0.001
Records mother’s BP at admission	46	93	0.007	< 0.001
Conducts PV examination only as indicated (4 hourly or based) on clinical indication	32	90	< 0.001	< 0.001
Performs hand hygiene	43	89	0.007	< 0.001
Provider identifies and manages severe Pre-eclampsia/Eclampsia	2	60	< 0.001	< 0.001
Initiates Partograph plotting once the Cx dilation is > = 4 cm	27	81	< 0.001	< 0.001
Provider interprets partograph correctly and adjusts care according to findings	15	51	< 0.001	< 0.001
**Just before pushing or at caesarean section**				
Preforms hand hygiene	49	96	0.002	< 0.001
Oxytocin within one minute of delivery of baby	59	96	0.05	< 0.001
Immediate newborn care	0	74	< 0.001	< 0.001
**Soon after delivery (within 1 h)**				
Delivers the baby on mother’s abdomen	42	95	0.002	< 0.001
Thermal management of newborn	15	84	< 0.001	< 0.001
Weighs the baby	88	99	0.67	0.048
Initiates breast feeding within one hour of birth	70	99	0.18	0.001
Provider identifies and manages Post-Partum Haemorrhage	4	59	< 0.001	< 0.001
Measures baby’s temperature	19	48	0.006	0.021
Records mother’ s temperature	12	48	< 0.001	0.002
**At the time of discharge**				
Counsels on danger signs to mother at time of discharge	26	65	0.002	< 0.001
Counsels on post-partum family planning to mother at discharge	43	98	0.001	< 0.001
Counsels on exclusive breast feeding to mother at discharge	59	83	0.001	0.001

^a^after adjusting for level of facility, average annual delivery load and resource availability for respective practice

### Delivery outcomes

**Fig. 1:**
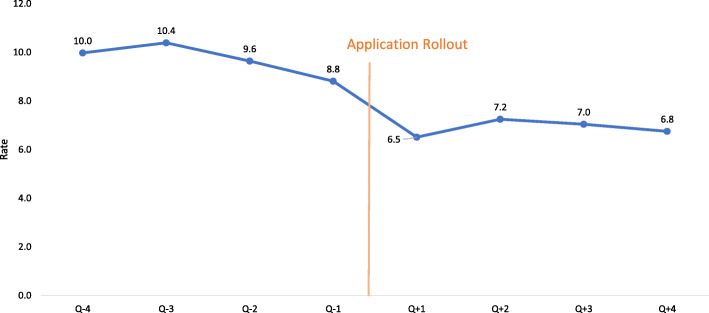
Trend of stillbirth rate (per 1000 live births) before and after roll out of ASMAN application

Figure 1 depicts the trend of fresh still birth rates in intervention facilities through the study period. There was a steady decline through the period and the difference between mean still birth rate of four quarters before intervention roll out and four quarters after intervention roll out, was statistically significant (*p* <  0.05).

The interrupted time series regression analysis on monthly data revealed that there was a decrease in fresh still birth rate after intervention roll out [− 0.92 (95% confidence interval: − 3.00 – 1.1.4)], though this change was not statistically significant. The trend over time did not change significantly either [0.12 (95% confidence interval: − 0.13 – 0.38)] (supplementary file 1).

**Fig. 2:**
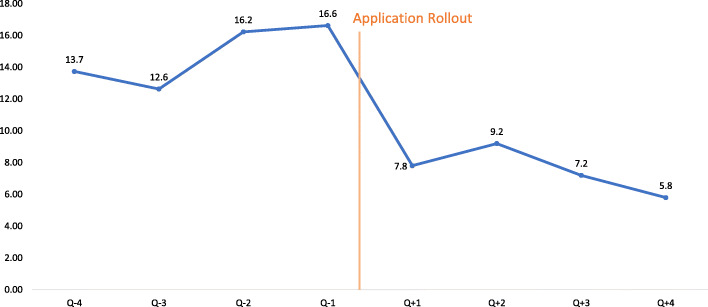
Trend of neonatal asphyxia cases (per 1000 live births) before and after roll out of ASMAN application

Figure 2 depicts the trend of neonatal asphyxia cases (per 1000 live births) in intervention facilities through the study period. There is a clear decline observed in number of cases after ASMAN application roll out. The difference between the mean number of cases in time periods before and after application rollout, found to be statistically significant (*p* <  0.05).

The interrupted time series regression analysis on monthly data revealed that there was a decrease in number of neonatal asphyxia cases after intervention roll out [− 3.12 (95% confidence interval: − 9.73 – 3.47)], though this change was not statistically significant. The rate of decline (slope) increased [− 0.36 (95% confidence interval: − 1.09 – 0.36)], though this too was not statistically significant (Additional file 1).

### Maternal complications

**Fig. 3:**
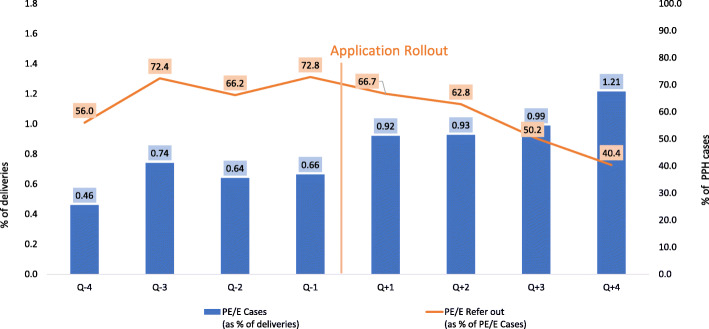
Trend of pre-eclampsia / eclampsia cases and referrals before and after roll out of ASMAN application

Figure 3 illustrates the trend of pre –eclampsia /eclampsia cases and refer out in intervention sites before and after ASMAN application rollout. There is an increase in identification of pre-eclampsia and eclampsia cases after application roll-out, while referral out declined over the same time period.

**Fig. 4:**
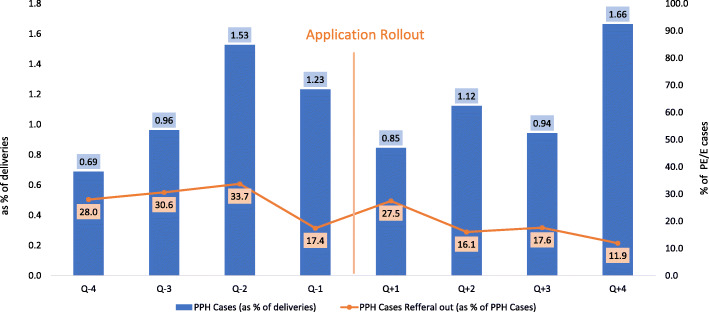
Trend of postpartum haemorrhage cases and referrals before and after roll out of ASMAN application

Analysis of postpartum haemorrhage cases and refer out trends in intervention sites before and after ASMAN application rollout revealed an increase in identification of postpartum haemorrhage cases and decrease in referral out (Fig. 4).

## Discussion

Our study described the uptake of ASMAN application among health care providers, the role of ASMAN application in improving adherence to key clinical practices, delivery outcomes and maternal complications. We have observed that, introducing ASMAN application resulted in statistically significant improvements in adherence to key clinical practices. However, overall completeness of case sheets in ASMAN application was low across all levels of facilities. We have observed reduction in fresh still birth rates and asphyxia, but these results were not statistically significant in interrupted time series analysis. On the other hand, our analysis showed that identification of maternal complications has increased over the period of program implementation and at the same time referral outs decreased.

### Uptake of ASMAN application

We have included completeness of case sheets in application as a measure of data quality as it is a commonly assessed dimension of data quality mentioned in a review [25]. Although, using medical records has been criticized for measuring quality of record keeping practices instead of quality of care, it was argued that patient case records should be part of quality of care assessment; because individual case records are essential not only for case management and peer review but also for assessing the impact of health interventions [26]. Additionally, it was noted that clinical record keeping enables continuity of care and improves communication between different health providers [27].

Our results indicated that the filling ratio during the post-partum period was low across all type of facilities. The poor clinical documentation was reported in a review of obstetric records conducted in India [28], with the extent of documentation varying between 1.3% (assessment of mother's condition at the discharge) and 99.1% (admission date). Similarly, a multicenter retrospective review of clinical records of cesarean delivery in five low-income countries identified poor-quality record keeping with missing information on key events, management of complications, and delivery outcomes [29]. Furthermore, we have found that the filling ratio across all pause points was low at the district hospital level and high delivery load facilities. In our qualitative study [30], health care providers mentioned that staff shortage, patient urgency, high caseloads prevented staff to complete all fields in the application. According to a study conducted by Chaturvedi [28] and colleagues, Madhya Pradesh state of India, documentation was better at district hospitals due to higher qualification of the staff in those facilities and availability of forms at the district hospitals. Although, the present study did not conduct comparison of documentation before and after ASMAN application roll out, due to the unavailability of data before application rollout, respondents in our qualitative study [30] reported that ASMAN application improved documentation. Additionally, a study conducted in Australian tertiary maternity facility [31] found that the use of electronic health records resulted in significant improvements in completeness of data captured. Furthermore, it was noted by the same study that the data captured electronically was easily available to providers compare to paper based records. Understanding the reasons for the low filling ratios at the PNC period would require further research. The low filling ratio at facilities with delivery load more than 300 could be explained by bigger client -provider ratio could be reasons as per anecdotal experience.

### Adherence to key clinical practices

Our study revealed that quality of care across all pause points improved during the implementation of ASMAN application. Furthermore, labor room staff in the qualitative study mentioned that ASMAN application improved their ability to take a complete history and physical exam, identify high-risk patients, manage cases confidently, facilitate provider communication, improve reporting processes, and ensure continuity of care for referral patients [30]. In our study we have observed that examinations around identification and management of complications, initiation of partograph, identification of post-partum haemorrhage increased significantly. These results indicate that ASMAN application has the potential to improve quality of care provided around the childbirth. These are very encouraging results, as timely identification of complications is crucial because over a third of maternal deaths, a significant proportion of pregnancy-related life-threatening conditions; approximately half of all stillbirths and a quarter of neonatal deaths are attributed to complications that occur during labour, childbirth or the immediate postpartum period [32–34]. Our findings are in line with the results of three systematic reviews on the role of CDSS use on providers’ adherence to clinical practices [12–14]. According to Garg and colleagues [12], better performance of providers was observed in studies where users were automatically prompted to use the systems compared with studies in which users were required to actively initiate the system. Additionally, the same review observed better performance in studies in which the trial authors also developed the CDSS software [12]. Moreover, a synthesis of high quality systematic reviews [14] showed that the positive effect of CDSS depends on the services which it was used; CDSS positively impacted providers’ performance in studies on drugs ordering and preventive care measures. Kawamoto et al., [13] identified four features strongly associated with a decision support system’s ability to improve clinical practice—(a) decision support provided automatically as part of clinician workflow, (b) decision support delivered at the time and location of decision making, (c) actionable recommendations provided, and (d) computer based. Additionally, CDSS should also provide periodic performance feedback, request documentation of the reason for not following system recommendations, and share decision support results with patients.

While there are studies on benefits of CDSS in general, there is very limited research on benefits of CDCC in maternity care. Duysburgh et al., [19] did not find a significant improvement in quality of antenatal and delivery care with the use of CDSS assessed in Burkina Faso, Ghana and Tanzania. They have concluded that, history taking, counselling, health education, laboratory investigations, and examination and monitoring of mothers and newborns during childbirth were not performed according to the standards. A study conducted in South Africa for improving compliance of health care workers with antenatal care guidelines found overall improvement, but it was not statistically significant [20]. McNabb et al., reports significant improvement in health counselling, technical services, quality of health education and patient satisfaction as a result of a mobile phone decision support during antenatal care in Nigeria [21].

Furthermore, studies conducted in India on the role of CDSS in the management of cardiovascular diseases [35] and hypertension [36] demonstrated positive role of CDSS in improving care, adherence to guidelines, counselling and follow up with patients that resulted in blood pressure reduction.

### Delivery outcomes and maternal complications

Improved practices around childbirth have a potential to prevent birth asphyxia and complications due to prematurity, which are some of the main causes of fresh still births and early neonatal deaths [34, 34, 37]. Our results revealed that there was a significant decrease in fresh still birth and asphyxia, however this decrease wasn’t statistically significant in interrupted time series analysis. These results are in line with the global evidence on the lack of evidence of potential impact of CDSS on patient outcomes [12–14, 37, 38]. Thus, according to the systematic review, majority of the studies assessed patient outcomes often without adequate statistical power to detect clinically important differences [12]. Jaspers and colleagues found only few studies on impact of CDSS on patient outcomes, though many of these have been too small in sample size or too short in time to reveal clinically important effect [14]. Furthermore, a systematic review and meta-analysis found that CDSS linked to electronic health records did not detect statistically significant reduction in morbidity and mortality [38].

The lack of statistically significant improvement in delivery outcomes could be attributed to the small sample size and short follow-up periods which is not long enough to assess the impact of CDSS on patient outcomes [14].

During the course of the program implementation, we have observed that identification of pre-eclampsia is increased while referrals for the same conditions decreased. This change could be attributed to the improved assessment at the time of admission. Moreover, we have observed the same trend in identification and referral of postpartum hemorrhage that could be explained by increased administration of oxytocin within 1 min of delivery.

### Strengths and limitations

The results of our study contributes to the dearth of literature on the role of CDSS in maternity care. From the onset of the program our team trained and worked closely with the focal point in each facility to ensure quality of data and timely reporting. Furthermore, as a part of program implementation, all providers were trained in distinguishing and reporting fresh still births. Additionally, data reported from facilities were regularly validated by our team. The availability of data before and during the program implementation allowed us to estimate the role of CDSS in improving quality of intrapartum care and delivery outcomes. Our study has few limitations. Firstly, it is possible that providers carried out the patient management according to the standards but didn’t record it in application. Secondly, our assessment involved direct observation of provider practices, as a result potential Hawthorne effect would have happened. To minimize Hawthorne effect we trained our project officers using standard observation checklist and standard operating procedures. Lastly, due to utilization of program data in our analysis, establishing causal claim is limited.

## Conclusion

To our knowledge, this is the first study that provides and evidence on the role of technology to improve the quality of intrapartum care and delivery outcomes in India. However, the results of this study could be generalized to another country with similar settings. Our study indicates CDSS has a potential to improve quality of intrapartum care and delivery outcome. Future studies with rigorous study design are required to understand the impact of technology in improving quality of maternity care. These studies could provide evidence on reducing the burden of maternal and neonatal death attributable to inadequate quality of care in India and globally.

## Supplementary Information

**Additional file 1.** Interrupted time series analysis on fresh still birth rates and incidence of neonatal asphyxia at intervention facilities.

## Data Availability

Qualified researchers may request data access by emailing the corresponding author. In such an event, the researchers will consult with the Government of Rajasthan and Madhya Pradesh state health departments before providing data access to the concerned parties.

## Abbreviations

ANM: Auxiliary Nursing Midwife
ASMAN: The Alliance for Saving Mothers and Newborns
CDSS: Computerized clinical decision support
GoI: Government of India
MO: Medical Officer
MSD: MSD for Mothers
OT: Operation theatre
PNC: Postnatal Care
SNCU: Sick Newborn Care Unit
SCC: Safe Childbirth Checklist
SPSS: Statistical Package for the Social Sciences
USAID: United States Agency for International Development
