# Supplementary material for: The role of digital clinical decision support tool in improving quality of intrapartum and postpartum care: experiences from two states of India
Source: BMC Pregnancy Childbirth. 2021 Apr 7;21:278. doi: 10.1186/s12884-021-03710-y (PMC8028806; doi:10.1186/s12884-021-03710-y)
Supplement: Supplementary file 1 — Additional file 1. Interrupted time series analysis on fresh still birth rates and incidence of neonatal asphyxia at intervention facilities. [file 12884_2021_3710_MOESM1_ESM.docx]

**Supplementary file 1: Interrupted time series analysis on fresh still birth rates and incidence of neonatal asphyxia at intervention facilities**

| Variable | Fresh still birth rate | | Neonatal asphyxia | |
| --- | --- | --- | --- | --- |
|  | Coefficient (SE) | 95% confidence interval | Coefficient (SE) | 95% confidence interval |
| _t | -0.16 | (-0.34 – 0.01) | 0.09 | (-0.55 – 0.74) |
| _x (change in intercept) | -0.92 | (-3.00 – 1.14) | -3.12 | (-9.73 – 3.47) |
| _x_t(change in slope) | 0.12 | (-0.13 – 0.38) | -0.36 | (-1.09 – 0.36) |
